# Supplementary material for: Proteomics-based clustering outperforms clinical clustering in identifying people with heart failure with distinct outcomes
Source: Commun Med (Lond). 2025 Nov 20;5:505. doi: 10.1038/s43856-025-01213-x (PMC12669581; doi:10.1038/s43856-025-01213-x)
Supplement: Supplementary file 2 — Supplementary Figs. [file 43856_2025_1213_MOESM2_ESM.pdf]

Proteomics-based clustering outperforms clinical clustering in identifying people with heart failure with distinct outcomes

Van Vugt *et al.*

Supplement

Content

Supplementary Figure 1……………………………………………………………………………2

Supplementary Figure 2……………………………………………………………………………3

Supplementary Figure 3……………………………………………………………………………4

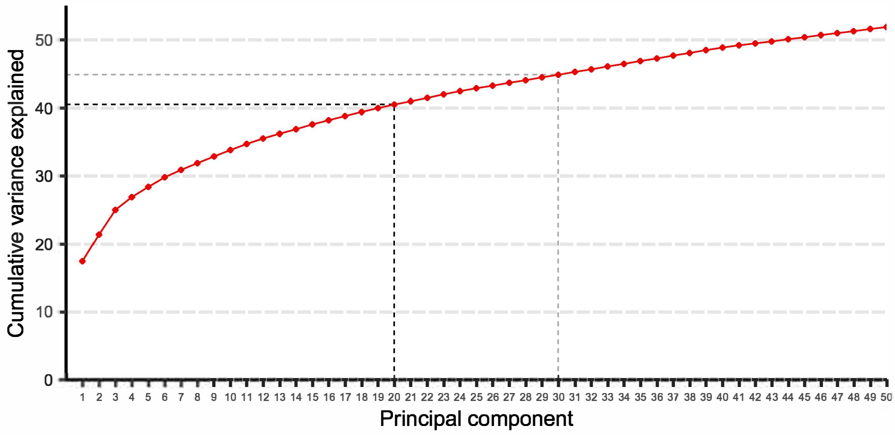

Supplementary Figure 1. Cumulative variance explained by the principal components of the proteomic clustering.
Principal component analysis was performed on 4,210 proteins in 379 BioSHiFT patients. Cumulative variance for 20 and 30 PCs is highlighted by the dashed lines.

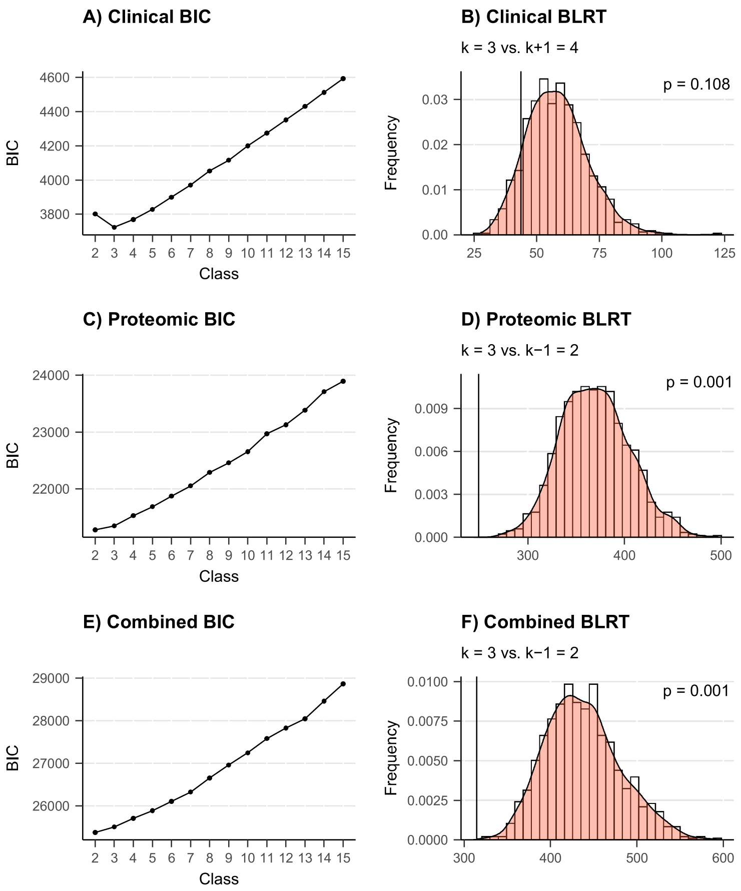

Supplementary Figure 2. Criteria for choosing the number of clusters.

The optimal number of clusters was defined using the Bayesian Information Criterion (BIC), depicted for A) clinical, C) proteomic, and E) combined clustering, and the log likelihood ratio distribution (BLRT), depicted for B) clinical, D) proteomic, and F) combined clustering. We compared the k cluster model with the lowest BIC with a k+1 cluster model calculating the likelihood ratio for the two models. The distribution of the likelihood ratio was estimated using 999 bootstraps, selecting the model with k+1 clusters based on a *p*-value of 0.05 or smaller. Vertical lines represent the observed log likelihood difference with which the p-values were determined. The *p*-value was obtained by comparing the observed difference to this distribution, using a one-sided test and no multiple testing correction.

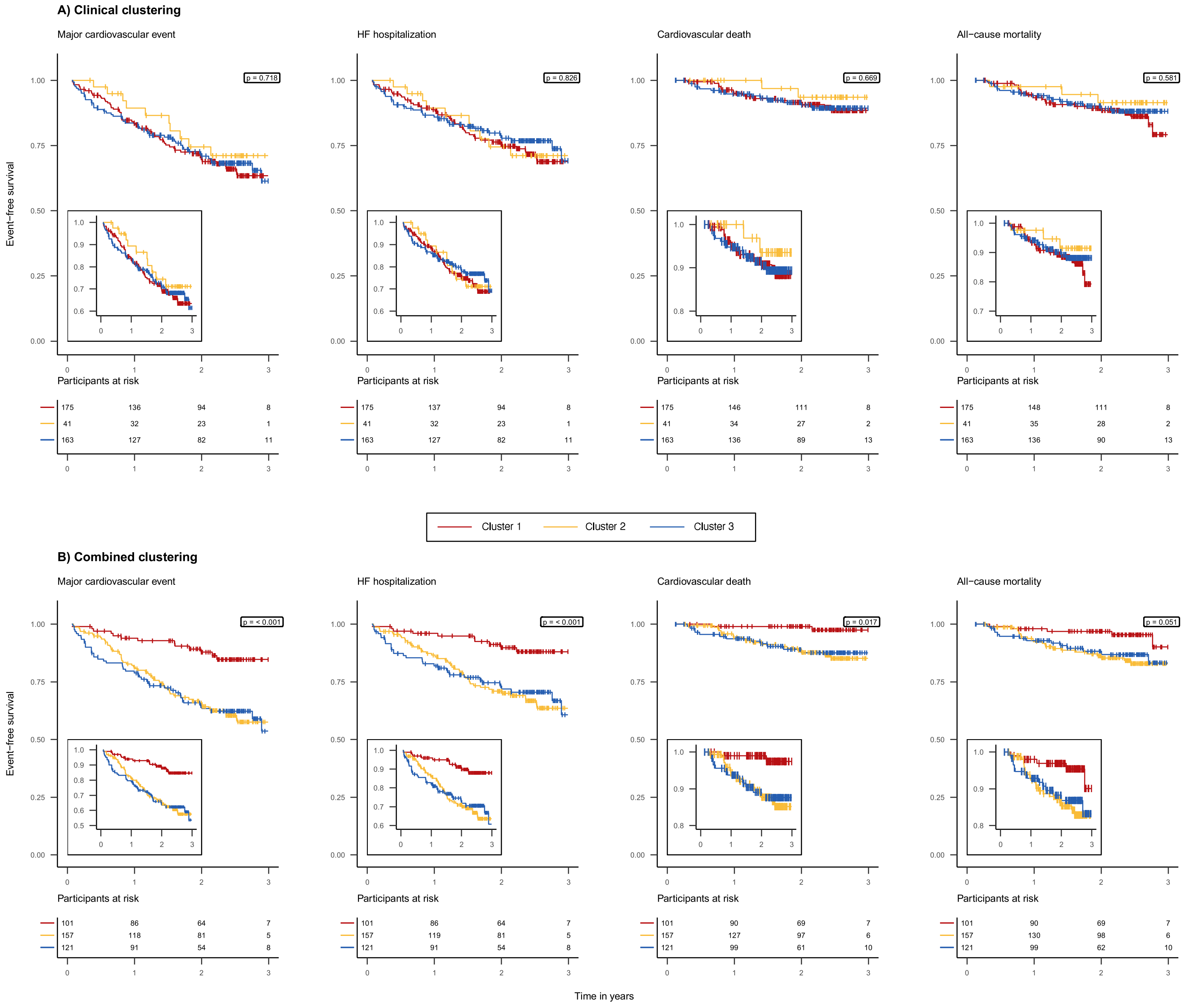

Supplementary Figure 3. Event-free survival per clinical outcome for the clusters.
Kaplan-Meier curve for the clinical outcomes stratified by A) clinical and B) combined clusters. Differences were assessed using the log-rank test. Abbreviations: HF = heart failure.
